# Students' self-repair in EFL classroom interactions: implications for classroom dynamics

**DOI:** 10.1186/s40862-022-00153-6

**Published:** 2022-07-25

**Authors:** Mohammed Beshir, Abiy Yigzaw

**Affiliations:** Bahir Dar University, Bahir Dar, Ethiopia

**Keywords:** Repair-strategies, Self-repair, Trouble-sources, EFL-classroom, Oral-interaction

## Abstract

This study aimed to evaluate whether or not students engage in self-help behavior when they encounter difficulties during their presentations. The participants were second year EFL college trainees; the data were obtained from their classroom presentations. The audio recorded data has been transcribed, coded, and categorized into quantitative and qualitative data. Descriptive statistical tools were employed to analyze the quantitative component, while thematic analysis was used to explain the qualitative data. Based on the findings, the students engaged in different kinds of strategies for self-repair, including same-information repair (repetition) 48 (36.9%); appropriateness repair 46 (35.4%); error repair 32 (24.6%); and back-to-error 4 (3.1%). From the point of view of the trouble sources that trigger self-repair, this research highlighted difficulties with syntactic and lexical errors as the most prevalent problems among EFL learners. The self-repair evidences revealed the importance of giving time for students to modify their utterances by themselves. In addition, the result could help teachers determine where to focus their efforts in helping students.

Interaction takes an important place in education and other avenues of life. It has become a vital tool of meaning-making, negotiating, and ensuring our involvement in constructing our immediate social world. Educational institutions aspire to develop interaction skills in students. Foreign language classes are settings where interaction competence is needed and practiced at the same time. English as foreign language (EFL) classes are one of the social worlds, practical interactional competence plays a vital role in successful teaching and learning (Walsh, 2011). This same social setting is where learners are expected to develop this competence while struggling to use it.

This interaction can increase learners' language storage and use all languages they possess (Allwright, 1984). To this end, Ohta (2001) notes that exchange facilitates acquisition because of the conversational and linguistic modifications that occur in such discourse and provide learners with the input they need. One of the primary forms of input in this process is the actions taken on learners' erroneous utterances. These actions, widely known as repair, are more than error correction and are defined as ways of addressing difficulties or troubles in speaking, hearing, and understanding that might happen in interaction (Schegloff, 2000).

Repair is universal in all talks (Schegloff et al., 1977). Research shows that language repair ensures smooth and accurate communication, and experience has shown that language learners can employ many repair strategies in second or foreign language interaction (Schegloff, 2000). The repair action takes a different form depending on who has taken steps to initiate it and resolve it. Finally, the success rate in resolving the communicative trouble varies across task types and communicative situations, requiring more exploration for a better understanding of the phenomena.

In light of the fact that previous research has not investigated self-repair strategies of Teacher Education Trainees in the Ethiopian context, it is imperative to examine this phenomenon to further substantiate the effects of output and related claims in SLA. The study examines how trainees at the College of Teacher Education repair their previous statements to make them more comprehensible.

## Review of related literature

In many social spheres, such as conversations with friends, conversations with colleagues, holiday events, and others, we depend on the ability to talk and understand others' talk. Of course, individuals are free to communicate when interacting with one another; however, a common phenomenon emerges when people do not regulate or modulate their communication breakdown. Therefore, interlocutors tend to use an interactional strategy called conversational repair (Schegloff et al., 1977). A study of the repair was first conducted on L1 speaker interactions, and then the findings were extended to analyze L2 speaker interactions. Schegloff et al. (1977) examined repair in everyday conversation comprehensively and systematically for the first time. They identified unspecified interrogatives, partial repeat, partial repeat plus question word, and understanding check as repair techniques native speakers use when they have conversation breakdowns. The study further revealed that the repairs focused more on the content and pragmatic errors than the linguistic errors. Thus, getting rid of communication barriers seems to be an entirely natural, ordinary, and normal thing to do in this situation.

Repair processes prevent communication breakdowns, and get the message across to the listener leads to second or foreign language learning (Rababah & Bulut, 2007). There have been relatively a few studies that consider the strategies students used to fix their inequities (Egbert, 1998; Liebscher & O'Cain, 2003). Egbert (1998) studied the different repair initiation skills exhibited in dyadic interviews by German college learners. She defines six types of repair initiations, namely the five types reported by Schegloff and colleagues (1977) in ordinary English conversation and an additional type that involves repetition. Liebscher and Dailey-O'Cain (2003) also examined data derived from a talk given by a German-language learner. A category of the repair replaced by Egbert's (1998) typology, which was a request for an interpretation, translation, or definition. They reviewed the repair patterns among students and their teachers and found that the repair schemes were different among the teachers and the students.

Likewise, Cho and Larke (2010) examined repair strategies of young learners and presented another classification. They identified nine types of repair strategies: unspecified, interrogatives, partial repeat, partial repeat plus question words, comprehension checks, requests for repetition, definition requests, translation requests, explanation requests, and nonverbal strategies. These repair strategies are applied to bridge the communication gap of students’ transition from their natural conversation to classroom conversation (Cho & Larke, 2010). Repair is a communication strategy used with interaction to modify, organize, and maintain communication (Rabab'ah, 2013). To help teach and develop students' communication skills, teachers should be aware of the repair techniques employed and interaction breakdowns.

Studies on repair identified different repair strategies such as self-initiation self-repair, other-initiation self-repair, other initiation other repairs, self-initiation-other repair (Schegloff, 2000; Wong & Waring, 2010). Among those, the current research investigated whether or not students used self-repair strategies to address communication problems. In this context, self-repair is defined as repairing communication barriers without external prompting. EFL learners could become aware of these tactics by understanding why and how they are being used, enabling them to converse with their interlocutors. In addition, the linguistic stock of learners is expanded when learners self-repair (Wong & Waring, 2010). Teachers who better understand how students deal with communication breakdowns and develop lessons that will help students improve their language proficiency gain greater insight into how they deal with communication breakdowns.

Self-initiation, the self-completed repair, is the preferred form of repair in daily conversations (Schegloff et al., 1977). What happens inside classrooms, however, tends to be very different from what happens in the daily conversations. For example, the process of repair in the teaching–learning context in general and in the EFL context, in particular, might not be as smooth as that of mundane context. In the EFL environment, communication breakdown becomes a widespread feature due to learners' dearth of a particular word or phrase or lack of the appropriate communicative strategies (Walsh, 2011); the rules students learn in class may disappear when they start talking. Most students interrupt what they have started to talk about instead of amending their gaps and continue talking. Such students may lose confidence to talk in front of people if they do this style repeatedly. On the other hand, some students might also continue talking without worrying about the rules they learn in class. Both cases might affect learners' communicative competence.

There are two principal classifications of self-repair: overt and covert repairs (Levelt, 1983). Overt repairs are further subdivided into same information repair (repeat), the different information repair (message replacement and fact repair), appropriateness repair (abandonment, replacement, and insertion repair), error repair and back-to-error repair **(**Kormos, 2000a, 2000b; Levelt, 1983; van Hest, 1996). In different information repairs, the speaker may decide to encode utterly different information from that which is currently being formulated in the phase of conceptualization, which is due to errors in the phase of conceptualization. In appropriacy repairs, however, the speaker feels that the statement needs to be clarified. Repairs on the former and later types involve modifying the first part of the sentence. The third class of repairs is error repair, where trouble occurs at the level of formulating the message, namely, an activated word is incorrectly activated, an inappropriate structure is adopted, or an incorrect phoneme is used. Levelt's repair classification emanates from the types of errors that students make in their interactions. When teachers observe such breakdowns in their students' language, they can spot linguistic issues and treat them accordingly.

Students can make conversational adjustments and language modifications when they actively attempt to turn information into useful information during the interaction. They could also check whether or not what they say makes sense and thus adjust their speech toward greater clarity and comprehension, which is considered the best method of learning the target language (Consolo, 2000; Ohta, 2001). This study takes a closer look at repairs initiated and completed by the same speaker of the trouble source. Self-repair is also indispensable to teachers to stay away from interference since students use tactics such as repetitions, restarts, I mean…, non-lexical perturbations (e.g., *uh*, *um*, *er*), a cut-off of an utterance or sound, pause (e.g., (0.3)), insertions, deletions, replacement and abandonment to resolve their difficulties (Wong & Waring, 2010). These techniques allow a speaker to think about the best way to address what they want to convey.

We cannot check students' minds, but we can hear what they say, see how they interact, use the target language, etc. This situation is where we have the opportunity to examine the classroom interactions for learning as a process. Knowing what is going on in classroom interaction is the same as studying educational theory (Ellis, 2000). Many studies (Ardini, 2015; Gass & Torrens, 2005; Hosoda, 2006; Robinson, 2006; Seedhouse, 2004; Shehadeh, 2001; Wong, 2000; and Wong & Waring, 2010) indicated the significance of repair routines for language learning. Also, Kormos (2000b) remarked that analysis of self-repair mechanisms can provide us with the most direct information about linguistic and psychological processes and the production and communication of the first and second languages. Nevertheless, it is not expected that it is common among learners since students have different language competence. Some learners might do well in reprocessing their output, but others may not. These processes need to be further studied for an accurate conclusion and for taking remedial actions.

As mentioned above, many studies have been conducted on the repair process in school settings. Nevertheless, in the Ethiopian context, the issue is not given attention. Only a few scholars have paid attention to repair processes in the classroom activities, but they have focused only on one of its components, i.e., error correction (Animaw, 2011; Birhanu, 2009; Samson, 2007; Abdissa, 2008; Emana, 1995; Tamirat, 1992). These studies revealed that everything the learner says is potentially subject to evaluation by the teacher. When what the learner says is not parallel with the teacher's pedagogical focus, error corrections are likely to be undertaken. As a result, classroom interaction's central phenomenon turns out to be error correction rather than repair. However, error correction alone cannot account for all the repair operations in the foreign language classroom.

It is expected that learners correct or modify their utterances to fix what seems erroneous to them. Kasper argues that "studies of repair in the foreign language classroom should include all repair activities rather than focusing on one specific repair type- the teacher's correction of learners' errors" (1985, p.200). Trying to focus exclusively on error correction causes learners to be informed of what is wrong and cannot figure out how to continue. Focusing only on error also disallows investigation of any difficulty occurring in the absence of error, including a learner's action on anticipated trouble. This study was initiated by the absence of studies in the area of repair in Ethiopia.

Students can be grouped into three categories in a classroom: low achievers, medium achieves, and high achievers. Their classroom performance in different activities varies as well. For example, in the repair processes, learners might reveal the following: failing to repair, expressing difficulty in repairing or communicating the intended meaning, appealing for help, repeating the trouble-source utterance without modifying, inserting new but not directly relevant information, switching the topic, or successfully reprocessing and reformulating the utterance (Shehadeh, 2001). These outcomes can be taken as evidence of learners' engagement. Language learners argue that they are much more likely to notice the differences between their interlanguage and the target language when a breakdown in communication leads to repair work (va Hest, 1996). This situation implies that if communication barriers are appropriately managed, they may not threaten teaching–learning. As stated, communication breakdown initiates repair strategies, but they are not always easy. Well-formed repair after self-initiation is considered successful. In light of this, this research is addressing the following research questions:

- What are the trouble sources that trigger students' self-initiated self-repair?
- What repair strategies do presenters use when they face trouble sources in their presentation?
- What is the most frequently used repair strategy?

## Method

The present study uses conversation analysis framework and is carried out as part of a descriptive qualitative method to describe repairs in the EFL classroom. The classroom interactions are genuine because they are specifically intended for teaching purposes and not for facilitating research. The qualitative approach was chosen to examine students' use of self-initiation self-repair strategies in a classroom presentation. A quantitative approach was also used to see simple totals and percentages of utterances and determine the classroom types of self-initiation strategies.

### Setting and participants

Amhara region is one of the administrative regions in Ethiopia. In this region, there are ten teacher education colleges in different zones. These colleges give training for three years to satisfy the needs of primary school teachers in the region. Woldia College of Teacher Education is one of these colleges; the study was conducted in this college. The college is chosen using simple random sampling-lottery method; it has six departments in various fields. The language department, which includes English and Amharic languages, is the focus of this study. Amharic is the official language of Ethiopia.

The participants of the study were second-year English major students. They range in age from seventeen to twenty, according to their roster. In terms of their gender, eighteen are females and fourteen are males. As their native language, each of them speaks Amharic. The students had been learning English as a subject beginning from grade one in primary school; they also used it as a medium of instruction starting from grade seven. Thus, before they joined college, they had learned English for ten years In addition, they had taken communicative English Skills I and II courses when they were first-year students in the first and second semesters, respectively. The courses are primarily offered to develop trainees' language skills to prepare them as excellent communicators in various activities. These indicated that the students have a shared experience in using English in various kinds of interaction situations. Two classes were attending their academic courses in the 2020–2021 academic year. One class was chosen from these two classes using a simple random sampling-lottery method to give both classes an equal chance. There were thirty-two students in the selected class.

### Procedures

The researcher first explained the study's general aim and built a good rapport with the instructors and students to conduct the research successfully. The researcher also observed the classroom for two periods during the teaching–learning process before conducting the data collection process. It was deliberately done to be more familiar to both the instructor and the students. This action was assumed to make the participants feel confident enough and do their classroom activities freely during data collection. The participants were orally asked for permission to audio record their classroom interactions. They gave full permission. After the recorded data were collected, the audio recordings were repeatedly viewed and listened to. Then, the audio data were transcribed.

### Task as data collection instrument

The use of the task shows it can be a successful tool in eliciting spontaneous self-repair from speakers. Classroom English is one of the courses in the second semester that students have to take. The course includes topics about self-introduction and introducing others, classroom management, greetings, starting a chat, taking turns to speak, and presentation skills. Among these tasks, data on students' self-repair were taken from their presentations, which was part of their college work. The teacher organized students into groups of five students. There were six groups; they shared ideas and chose their topic for the presentation, and the teacher has confirmed that the topics fall within students' competence.

The teacher also explained that students had to follow specific procedures. These include eye contact, audibility, fluency of speech, an outline of what they planned to discuss, and a conclusion. Before the presentation, the teacher gave one day for preparation. The teacher allowed 7–10 min for the presentation. The chosen student from each group presented what they discussed in their respective group on the presentation day. Students' presentations were captured using an audio recording device. The recording lasted a total of 52 min. The topics were the following: (a) The social, political and economic impacts of covid-19, (b) The current situation of Ethiopia, (c) Great Ethiopian Renaissance Dam (GERD), (d) Tourist sites in the Amhara Regional State, (e) Ways of improving speaking skills, and (f) Education.

### Analysis

The recorded data were analyzed both quantitatively and qualitatively. The overall transcribed data showed that the six presenters uttered 653 utterances. In total, 130 self-repair strategies were identified in the data. They can be divided into four types: same information repair (repetition) 48 (36.9%); The appropriateness repair category (replacement, insertion, and abandonment) covered 46 cases (35.4%); in this group, replacement, insertion, and abandonment each occurred 30, 6, and 10 times respectively; error repair 32 (24.6%); and back-to-error 4 (3.1%). The detailed qualitative analysis of the transcribed data is also presented below.

#### Extract 1

"How we improve English speaking skills" was the topic presented by the trainee. Despite its full transcription, the student's utterance is not included since this part of the speech clearly showed the student's use of repair. Most of the presenter's repetition was based on different word categories.

S1: Today I will I will give a short presentation on on how umm on how to to to improve English.

speaking skills. It is very very essential skill. So:: we will focus on the key STEPS

that help that help us umm improve English speaking skill. Okay, (.) the skill the skill is

important. Okay my name is Abinet Yalew by the way. I did not tell you, my name. I am

SORRY for that...

The student repeated prepositions (on, to), the definite article (the), verbs like (help, will), the pronoun (I), adverb (very) for different purposes. The repetition phenomenon prevailing in this excerpt indicated that the student used it to search for a word or idea. This strategy helps the speaker to lengthen the time to find out the correct words he wants to mention. The student also used repetitions to give emphasis. For example, in the part of the utterance "It is very very essential skill" the adverb '*very'* is repeated. Here the adverb 'very' is used to magnify the importance of the skill.

The researchers recorded repetition when individuals repeat a particular language item by delaying the next lexical item or gaining time to retrieve the next complex lexical item. In the present study, repetitions of nouns, personal pronouns, conjunctions, prepositions, definite and indefinite articles, and demonstrative pronouns were observed. In most cases, speakers use repetition as a strategy to get time before delivering the next lexical item (Sato, 2012). In addition to delaying the next lexical item, repetition is also vital for emphasizing (Bada, 2010). This purpose of repetition is also observed in the data.

#### Extract 2

The trainee addressed a current issue 'The social, political and economic impacts of Covid -19'. To correct her inaccurate statements, she employed multiple self-repair strategies, such as repetition and error correction. Although she noticed some errors in her utterances and fixed them, other errors went unnoticed.

S2: Today, I am gonna give you a short presentation on on the IMPACTS of umm the impacts

on social, political and economic impacts of covid 19 covid 19. Schools schools have been

closed as you know. Celebrations have been cancelled. We were we were keep (.) we were

keeping we were keeping our physical distance from our parents. The elections sorry (.) the

election has been delayed. The delayed election umm because of this disagreement occurred

among the ruling the ruling party and some opponent parties. That is all we have on social

impacts. The other is economy impacts…

The presenter started her utterance with a syntactical error. She did not repair it. She also made a wrong tense form as "were keep," but she realized that her verb tense was incorrect. In this case, she immediately repaired it as "were keeping" after spotting the error. She repeated a preposition 'on' to gain time to retrieve the next lexical item.

Again, the presenter used the plural noun "elections" instead of using the singular form. She used the expression '*sorry'* to indicate to her audience that the plural form of the word "election" is not appropriate here. So, she immediately repaired it by using the singular form "election." Here, in the same extract, the presenter started a sentence by saying, "But, the delayed election…" the presenter did not continue with what she started because she became aware that the syntactic error leads her to an impasse then, after hesitation, pause "*umm*" she started the sentence with a different syntactic structure and repaired. She also articulated the sentence '*The other is economy impacts.'* At this time, the student used the noun 'economy' instead of using the adjective' economic.' She did not repair this lexical error. All error types might not be repaired. This situation by itself is essential to the teacher to identify areas of support.

Presenters made errors while they were presenting. They became aware of some of the errors they made and corrected them. However, some kinds of errors were also left as they were. These errors might have resulted from their lack of awareness. Such kinds of outputs are signs for teachers where to focus on for further support.

#### *Extract 3*.

At the beginning of the presentation, the presenter pleaded with his classmates to lend him their ears. In order to correct his utterances that he considered inaccurate, he used repair strategies such as repetition, error repair, and replacement. As part of the repair process, some lexical items were replaced without repair being initiated.

S3: I want you to listen to me. I tried I try to speak a bit slower. My presentation focuses on on

on the current situation of of our country. As we KNOW, our country is in political reform. (.)

… but it caused war I mean chaos among ethnic groups in some regions. For example, in

Oromia and Benishangul and war between TPLF party and the prosper- government forces.

I think I believe the current situation of our country is not good. Many people are displaced

umm displace from their home…

In extract (3), the student was notifying students to give him their ears. He also informed his classmates how he presents in terms of his voice quality. He used past tense "tried" instead of the bare infinitive, and then he repaired it as "try". The student realized that the past form indicated completed actions, and he also realized that his presentation is on the way to be presented. So, he did the successful repair. He also replaced the word 'think' with 'believe' without using any initiation strategy. He preferred the word believe instead of the word think. Based on their similarity, the repair might have been only a word change without any significant change to the concept.This replacement strategy has not resulted from an error; instead, it is a matter of word choice.

The student used the self-editing expression "I mean' to inform his classmates that he would reformulate his prior utterance. The initiator 'I mean' is used in English conversation to announce in the context of replacement or abandonment of the already-manufactured turn-constructional unit (Rieschild, 2011). The word 'I mean' is used in many ways: explicating, correcting, creating narrative tension, and reserving turn (Rieschild, 2011). The expression 'I mean' is also used in Amharic speakers with its equivalent meaning to address communication barriers and control the stage. So, using the expression as a strategy might not be new to the students.

The presenter also uttered the sentence as *'war between TPLF party and the prosper- government forces.'* He started with the trouble-causing word (possibly prosperity), only to be cut off and replaced by the noun " government." Here, the student stopped his utterance by cutting it off just after the second syllable '*prosper-,'* marked with a dash at the end of the syllables. He did not use any lexical resource to indicate that his first attempt was wrong. The cut-off is one of the self-initiators that speakers use to interrupt their ongoing utterance when they face something inappropriate.

The presenter also mentioned the other problem Ethiopia faced as 'many people are displaced umm displace from their home…'. Here the student formulated correct expression in the first attempt. However, he assumed it was improper and repaired it; he changed the appropriate utterance with the inappropriate ones in this case. This case indicated that there might be possibilities that students shift from the appropriate utterance to the inappropriate ones. It is also the input for teachers to emphasize where to focus on. This error indicated a lack of clarity in the form of active and passive voice. This situation is termed as back to error (van Hest, 1996).

In extract 3 above, in the sentence "… but it caused a war, I mean chaos among ethnic groups in some regions." The presenter recognized that the word *'war'* is inappropriate for the idea he wanted to transfer. Then, he took time using the signal '*I mean'* and substituted the noun 'war' with the appropriate word 'chaos' to the situation. He might assume that 'war' is very worst compared to the situation. So, he preferred 'chaos'. It is a successful repair. He also replaced 'prosperity' with 'government forces' without forewarning to let his addressees become aware of the turn's suspension. Such a form of repair sometimes might create confusion upon listeners. The choice of words or expressions is the crucial aspect of this technique. The speaker tended to replace words with new suitable ones when he realized that the words such as the ones he used before are not suitable.

In self-initiated repair, replacement is a typical structure (Emrani & Hooshman, 2019). A speaker replaces a word or an utterance with new ones considered appropriate concerning the previous utterances in this strategy. Both the previous and the newly changed segment might have the same discourse structure. The following data revealed this case.

#### Extract 4

Introduction of the topic was the trainee's first step in his presentation. This topic is a hot issue in Ethiopia. He employed self-repair strategies such as repetition and replacement. Upon transcribing his utterances, it is evident that he attempted unsuccessfully to repair the problem. A student's unsuccessful efforts give the instructor insight into where further support is needed.

S4: My presentation is about Ethiopian great renaissance DAM. The dam is is the BIggest

project in the country. The dam is built umm the Ethiopian people is constructing the dam

to generate electric power. It creates umm it produced created 6000 megawatts.

The presenter started the sentence with 'the dam is built,' but he thought that the way he started his idea is not the way he intended. So, he rephrased the sentence using *'The Ethiopian people is constructing the dam…'* This instance is a concrete example of message replacement repair (Levelt, 1983). This example could be regarded as a communicative strategy. If the participants feel that they cannot continue, they can choose other ways to express themselves. Again, in the sentence *'It creates umm it produced 6000 megawatts', the student repaired.* In this case, he changed the verb '*creates'* to '*produced,'* but, not successful. The student used wrong tense. From this example, it is possible to deduce that every repair might not be successful. It is clear evidence to teachers where to focus for further support.

Abandonment means that the speaker of the repaired turn aborts or stops completing what he or she is talking about, and then he or she restarts a new turn (Ardini, 2015). It is a self-interruption strategy where the speaker thinks that they are diverting from the main topic or what they are trying to say is unimportant to the listener.

#### Extract 5

The presenter introduced the issue that she and her group discussed. As she took responsibility for presenting the issue, she informed her classmates. In her presentation, she repaired the prior utterances that she thought were erroneous using self-repair strategies. She used cut-off to initiate repair and apply replacement as a self-repair strategy.

S5: Our group discussed education. I am going to sa-present it. Umm education is necessary for

development. It is gro-divided into formal and informal. formal education give umm is

given in schools. Informal education can be gained in life experience. Formal education is

started in our country hundred years ago …

Here, the presenter stopped her utterance by cutting it off just after the syllable' *sa-'*and '*gro-'*marked with a dash at the end of the syllables. These cut-offs function as a repair initiator. After that, she recycled the word form and replaced "*sa-"* with 'present' and *"gro-"* by 'divided'. She did not use any lexical resource to indicate that her first attempt was wrong. Instead, she cut off what she thought erroneous and immediately substituted it with what she thought acceptable.

In the utterance, ‘umm education is necessary for development’, the student started her utterance with the non-lexical initiator *'umm,'* but the strategy did not show any semantic value*. It merely functions* as a discourse marker. Again, the student uttered 'formal education give umm is given in schools', the verb (*give*) is a trigger, and (*um*) is used as a self-initiating strategy. The student detected that the form of the output (*give)* was erroneous, stopped the speech flow, and finally corrected the error by replacing the verb (*give*) with the present passive form of the verb (*is given*). After becoming aware of the error, she did not automatically break her utterances; instead, she used the editing signal (*um*) until she substituted the appropriate verb form. Finally, she succeeded systematically without creating a gap between her and the recipients. She informed when formal education was started in Ethiopia; in this case, she committed tense error. She did not correct it.

#### Extract 6

The trainee introduced the topic of his presentation. Like other presenters, this presenter also used self-repair strategies such as insertion and repetition. As we observe in the extract, both strategies were not used to treat errors rather they were incorporated to give more clarity.

S6: My presentation is about the tourist sites in AMHARA region. In our reg::ion, there are

many tourist sites, (.) umm many many attractive tourist sites.

In extract 6, the presenter presented about tourist sites. "there are many tourist sites *umm* many many attractive tourist sites" In this case, it is not the error that triggered the use of the non-linguistic signal (*umm*). There is no observed correction in this part of the utterance; instead, the speaker used the signal (*umm*) to repeat part of the original utterance and inserted the adjective *attractive* to specify his message. The presenter is not satisfied with his first utterance. He might want to describe tourist sites by inserting the adjective *'attractive'* before the noun tourist sites. Inserting is also used as a strategy of self-initiated repair structure. Students who used this strategy repeated the previous utterance and inserted a word or an utterance to specify the information.

## Discussion

The primary objective of this study was to identify the self-repair strategies used by the trainees in their classroom oral presentations. It is of value for both teachers and learners to understand how trouble sources are managed in a language classroom since it helps them manage miscommunications and breakdowns in communication (Seedhouse, 2004). As indicated in the result section, the recorded data contain nearly all of the features of self-initiated strategies of repair. This result is also compatible with earlier study findings (Hosoda, 2006; Kormos, 2000a, 2000b; Shehadeh, 2001). Based on the results, the self-initiated repair was used when the speakers encountered problems with retrieving the target language item. As the data indicated, most of the students' repair processes were successful. Learners would be more likely to produce well-formatted output if they initiated self-repair efforts (Sato, 2012).

The data vividly showed that same information repair (repetition) makes up the highest percentage of repairs (36.9%). The speakers repeatedly used repetition to gain time to recall the next lexical item. In repetition, a speaker might avoid making mistakes; this is a communicative strategy. It is used in repeating one or two words. Thus, repetition can remind a listener/s of the importance of the coming utterance. It was evident that the participant's repetition strategies enabled them to gain more time while still maintaining a language environment conducive to meaningful English language discussion. Rieger (2003) states that repetition, a type of self-repair, features a particular sequence of repair strategies where the repairable and repairing lines co-occur initiator of the repairable performs the repair.

The second-largest type of repair was appropriateness, accounting for (35.4%). A repair of appropriateness concerns how thought is expressed clearly and properly, Replacement, insertion and abandonment are specific repair techniques in this category. Speaker used replacement when recognizing that what they had said before is not the right word(s). Therefore, they tend to interrupt themselves with appropriate replacements. In inserting, speakers inserted their words and utterances in a repaired segment. It has also been observed that the repaired segment is repeated throughout. The speaker then repeats the last line of speech and inserts a new element, such as a word or utterance. Likewise in abandonment, the data revealed that the speakers stopped completing what they articulated, and instead, they restarted a new turn set. It is a self-interrupting strategy in which the speaker believes that the listener does not care about deviating from the topic during their presentation.

The third type of repair observed in the transcribed data was error repair, representing (24.6%) of all repairs. Among their errors, the presenters repaired lexical and grammatical errors. The most common of these error repairs were lexical, which was closely followed by grammatical repairs. The other type, back-to-error account for the lowest proportion (3.1%). This situation is interesting because sometimes the speaker says something correctly, but then changes it into an incorrect statement.

Non-lexical initiators incorporate quasi-lexical fillers (uh, er, umm), cut-off, and lengthening sounds (Schegloff et al., 1977). Quasi-lexical fillers are said to be editing terms (Levlet, 1983). In the present data, a distinction was observed between editing terms with and without semantic value. Editing terms without semantic value, such as 'um' and 'er', are empty fillers that merely function as discourse markers. Such a case is reflected in the data. Editing terms with semantic value can be described as fillers semantically related to the repair under construction. They indicate the kind of linguistic trouble the speaker is experiencing and help the listener interpret the repair. The cut-off is also one of the self-initiators that speakers use to interrupt their ongoing utterance when they face something inappropriate immediately.

## Conclusion

An analysis of learners' self-repair can provide insights into their general perceptions and conceptualizations of the target language, their areas of difficulty, and their language acquisition strategies and attitudes. The pedagogical implications also include language teachers' awareness of the role that repair organizations can play in facilitating learning opportunities. The present study results confirm that learners use different types of self-initiation self-repair strategies for different purposes. Repair strategies can facilitate and promote fluency, accuracy, and effectiveness especially during student centered activities. In order to reduce the frequency of errors or mistakes, teachers should include more fluency practices for their students.

Nevertheless, lack of linguistic knowledge may impede them from applying repair practices or extend the time the repair will take. Students with speech difficulties need help from EFL teachers by receiving constructive feedback about their speech. As the data further indicated, all repairs might not be successful. Some instances of utterances were repaired wrongly. Such kinds of data are also essential for teachers as input to identify learners' gaps. In a foreign language education context, like Ethiopia, it is indispensable to give students opportunities to practice using the language because their chance of using the language outside the classroom is limited.

## Data Availability

Not applicable.

## Abbreviations

EFL: English as a foreign language
L1: First language
L2: Second language

## References

[CR1] Abdissa, S. (2008). ELT teachers’ corrective feedback on students’ oral errors [Unpublished M.A thesis]. Addis Ababa University.

[CR2] Allwright R (1984). The importance of interaction in classroom language learning: A brief historical overview. Applied Linguistics.

[CR3] Anteneh, A. (2011). Oral corrective feedback: an exploratory case study of the interplay between teachers’ beliefs, classroom practices, and rationales [Unpublished doctoral dissertation]. Addis Ababa University.

[CR4] Ardini SN (2015). Repair strategies of teacher's talk in EFL classroom. ETERNAL (English Teaching Journal).

[CR5] Bada E (2010). Repetitions as vocalized fillers and self-repairs in English and French interlanguages. Journal of Pragmatics.

[CR6] Birhanu, M., Bekana, A. (2009). EFL teachers’ treatment of students’ oral errors in EFL classrooms: Shamboo Senior Secondary and Preparatory School (Grade 9 in focus) [Unpublished M.A Thesis]. Addis Ababa University.

[CR7] Cho EH, Larke P (2010). Repair strategies usage of primary elementary ESL students: implications for ESL teachers. Tesl-Ej.

[CR8] Consolo D, Hall JK, Verplaetse LS (2000). Teacher's action and student oral participation in classroom interaction. Second and foreign language learning through classroom interaction.

[CR9] Egbert M, Young R, He W (1998). Miscommunication in language proficiency interviews of first-year German students: A comparison with natural conversation. Talking and testing: Discourse approaches to the assessment of oral proficiency.

[CR10] Ellis R (2000). Task-based research and language pedagogy. Language Teaching Research.

[CR11] Emana, T. (1995). Teachers’ corrective treatment of Grade 11 students’ oral errors in the English language classrooms [Unpublished M.A thesis]. Addis Ababa University.

[CR12] Emrani, F., & Hooshman, M. (2019). A conversation analysis of self-initiated self-repair structures in advanced Iranian EFL learners. *International Journal of Language Studies,**13*(1), 57–76.

[CR13] Gass SM, Torres MJA (2005). Attention when?: An Investigation of the ordering effect of input and interaction. Studies in Second Language Acquisition.

[CR14] Hosoda Y (2006). Repair and relevance of differential language expertise in second language conversations. Applied Linguistics.

[CR15] Kasper G (1985). Repair in foreign language teaching. Studies in Second Language Acquisition.

[CR16] Kormos J (2000). The timing of self-repairs in second language speech production. Studies in Second Language Acquisition.

[CR17] Kormos J (2000). The role of attention in monitoring second language speech production. Language Learning.

[CR18] Levelt WJM (1983). Monitoring and self-repair in speech. Cognition.

[CR19] Liebscher G, Dailey-O’Cain J (2003). Conversational repair as a role-defining mechanism in classroom interaction. The Modern Language Journal.

[CR20] Ohta AS (2001). Second language acquisition processes in the classroom: Learning Japanese.

[CR21] Raba'ah G, Bulut D (2007). Compensatory strategies in arabic as a second language. Poznan Studies in Contemporary Linguistics.

[CR22] Rabab'ah G (2013). Strategies of repair in EFL learners' oral discourse. English Language Teaching.

[CR23] Rieger CL (2003). Repetitions as self-repair strategies in English and German conversations. Journal of Pragmatics.

[CR24] Rieschild V (2011). Arabic yacni: Issues of semantic, pragmatic, and indexical translation equivalence. Intercultural Pragmatics.

[CR25] Robinson JD (2006). Managing trouble responsibility and relationships during conversational repair. Communication Monographs.

[CR26] Samson G/Hiwot. (2007). An investigation of teacher corrective feedback to student oral errors in the EFL classrooms [Unpublished M.A thesis]. Addis Ababa University.

[CR27] Sato, R. (2012). Self-initiated Self-repair Attempts by Japanese High School Learners While Speaking English. *BRAIN. Broad Research in Artificial Intelligence and Neuroscience*, *3*(2), 17–28. Retrieved from https://www.edusoft.ro/brain/index.php/brain/article/view/359/403

[CR28] Schegloff EA (2000). Overlapping talk and the organization of turn-taking for conversation. Language in Society.

[CR29] Schegloff EA, Jefferson G, Sacks H (1977). The preference for self-correction in the organization of repair in conversation. Language.

[CR30] Seedhouse P (2004). The organization of turn-taking and sequence in language classrooms. Language Learning.

[CR31] Shehadeh A (2001). Self-and other-initiated modified output during task-based interaction. Tesol Quarterly.

[CR32] Tamirat, W. (1992). Classroom feedback behavior of grade eleven English teachers. [Unpublished M.A thesis]. Addis Ababa University.

[CR33] van Hest E (1996). Self-repair in L1 and L2 production.

[CR34] Walsh S (2011). Exploring classroom discourse: Language in action.

[CR35] Wong J (2000). Delayed next turn repair initiation in native/non-native speaker English conversation. Applied Linguistics.

[CR36] Wong J, Waring HZ (2010). Conversation analysis and second language pedagogy: A guide for ESL/EFL teachers (First).

